# Breast Cancer Metastasis to the Stomach That Was Diagnosed after Endoscopic Submucosal Dissection

**DOI:** 10.1155/2016/2085452

**Published:** 2016-11-09

**Authors:** Masahide Kita, Masashi Furukawa, Masaya Iwamuro, Keisuke Hori, Yoshiro Kawahara, Naruto Taira, Tomohiro Nogami, Tadahiko Shien, Takehiro Tanaka, Hiroyoshi Doihara, Hiroyuki Okada

**Affiliations:** ^1^Department of Gastroenterology and Hepatology, Okayama University Graduate School of Medicine, Dentistry, and Pharmaceutical Sciences, Okayama, Japan; ^2^Department of Breast and Endocrine Surgery, Okayama University Hospital, Okayama, Japan; ^3^Department of Endoscopy, Okayama University Hospital, Okayama, Japan; ^4^Department of Pathology, Okayama University Hospital, Okayama, Japan

## Abstract

A 52-year-old woman presented with stage IIB primary breast cancer (cT2N1M0), which was treated using neoadjuvant chemotherapy (epirubicin, cyclophosphamide, and paclitaxel). However, the tumor persisted in patchy areas; therefore, we performed modified radical mastectomy and axillary lymph node dissection. Routine endoscopy at 8 months revealed a depressed lesion on the gastric angle's greater curvature, and histology revealed signet ring cell proliferation. We performed endoscopic submucosal dissection for gastric cancer, although immunohistochemistry revealed that the tumor was positive for estrogen receptor, mammaglobin, and gross cystic disease fluid protein-15 (E-cadherin-negative). Therefore, we revised the diagnosis to gastric metastasis from the breast cancer.

## 1. Introduction

Breast cancer can metastasize to local or distant locations, and the common locations for extramammary metastasis are the bones, lungs, liver, soft tissues, and adrenal glands [1, 2]. However, breast cancer metastasis to the gastrointestinal tract is uncommon, and the diagnosis of gastrointestinal metastasis or carcinomatosis can be complicated by its infrequency and its morphological resemblance to primary gastrointestinal neoplasms [1, 3].

Herein we report a patient with breast cancer who underwent radical mastectomy and developed a gastric lesion 8 months later. The gastric lesion was initially diagnosed as primary gastric cancer, based on the endoscopic features and histopathological findings. However, histological reassessment of an endoscopically resected specimen ultimately allowed us to reach the correct diagnosis of gastric metastasis from the primary breast cancer. This case highlights the importance of considering gastric metastasis of breast cancer as a differential diagnosis in patients who present with a gastric lesion and a history of breast cancer.

## 2. Case Presentation

**Figure 1:**
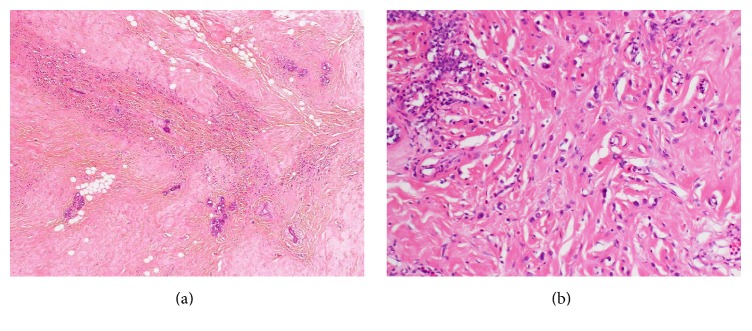
Histological analysis of the left breast. The initial diagnosis was scirrhous carcinoma, because the tumor cells were associated with a dense connective tissue in the stroma. The diagnosis was later revised to invasive lobular carcinoma.

A 52-year-old woman was diagnosed with stage IIB primary breast cancer in the left breast (cT2N1M0). On immunohistochemical examination, the tumor was positive for estrogen receptor (ER) but was borderline (2+) for human epidermal growth factor receptor 2 (HER2) expression. Fluorescence* in situ* hybridization revealed that the tumor cells were negative for* HER2* amplification. The patient was treated via neoadjuvant chemotherapy with epirubicin and cyclophosphamide, followed by paclitaxel. After the chemotherapy, computed tomography and magnetic resonance imaging revealed that the tumor was still present in patchy areas. Therefore, we performed modified radical mastectomy and axillary lymph node dissection for the left breast cancer. Histological analysis revealed that most of the mammary tissue was hyalinized, due to the effect of the neoadjuvant chemotherapy, and only a few viable carcinoma cells were present. The postsurgical histological diagnosis was scirrhous carcinoma of the left breast that had metastasized to seven lymph nodes in the axillary region (Figure 1). The cancer cells were positive for ER, partially positive for progesterone receptor (PgR), and negative for HER2 expression (1+). Therefore, we started tamoxifen therapy after the surgery, with radiation (50 Gy) of the entire affected area.

**Figure 2:**
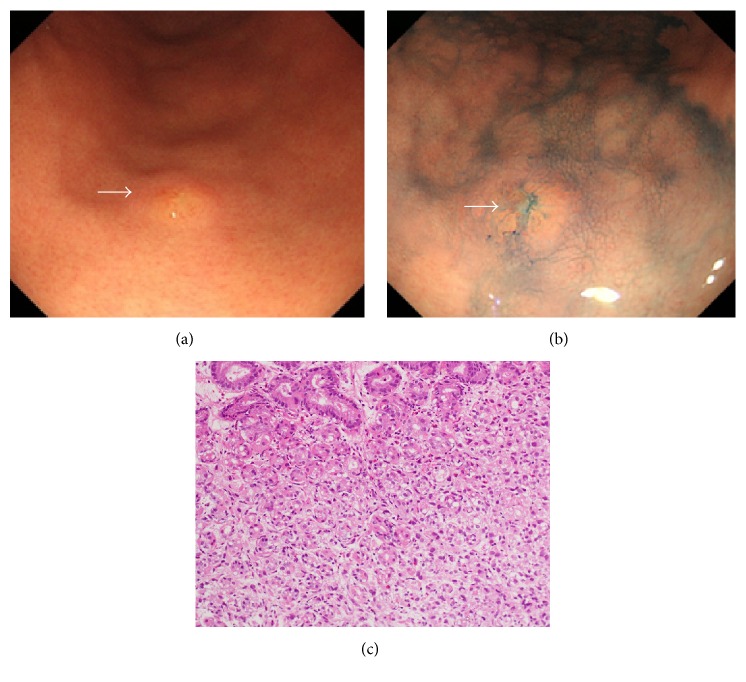
Esophagogastroduodenoscopy reveals a 0-IIc lesion with a size of 4 mm on the greater curvature of the gastric angle.

**Figure 3:**
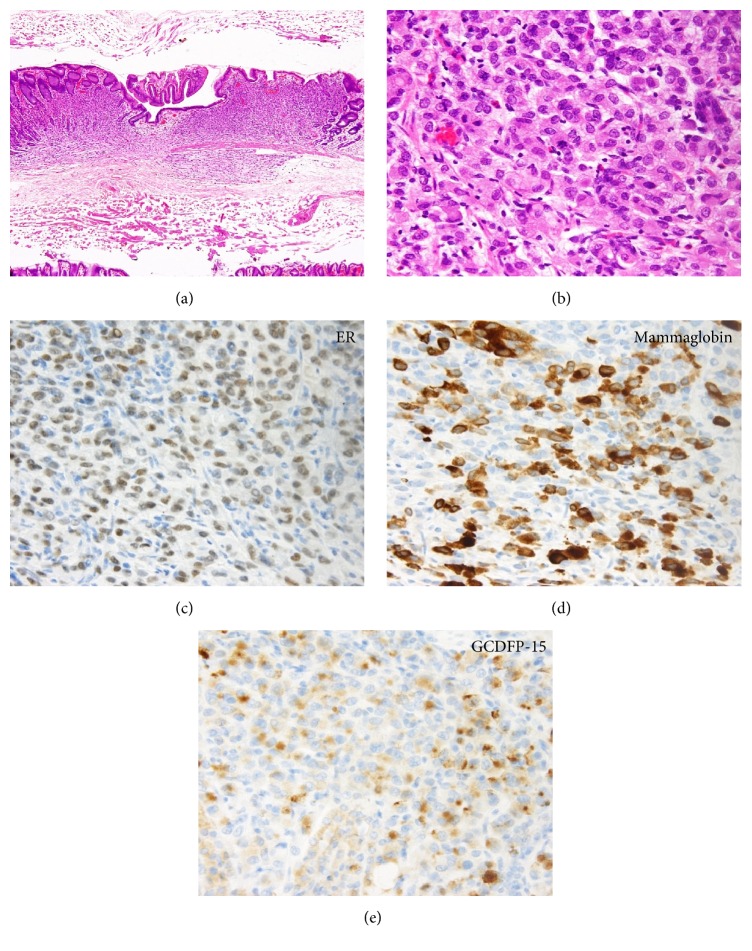
Histopathology of the tumor, which was localized within the mucosal layer to the submucosal layer.

At 8 months after the surgery, the patient underwent esophagogastroduodenoscopy for a general health check-up. The endoscopic examination revealed a slightly depressed lesion with a size of 4 mm on the greater curvature of the gastric angle (Figures 2(a) and 2(b)). Histological evaluation of a biopsy specimen revealed proliferation of signet ring cells in the proper mucosal layer of the stomach (Figure 2(c)). Destruction of the fundic glands by the cancer cells was also observed, although atypical cells were absent from the epithelium. Therefore, based on a diagnosis of primary gastric cancer, endoscopic submucosal dissection was performed. Histopathological examination of the resected specimen revealed that the tumor cells were confined to the mucosal and submucosal layers (Figures 3(a) and 3(b)). Immunostaining revealed that the tumor cells were positive for ER (Figure 3(c)), mammaglobin (Figure 3(d)), and gross cystic disease fluid protein-15 (GCDFP-15) (Figure 3(e)) and were negative for E-cadherin. Therefore, the diagnosis was revised to gastric metastasis from the primary breast cancer. The type of the primary breast cancer was also revised to invasive lobular carcinoma (rather than scirrhous carcinoma), based on the morphological and immunophenotypic characteristics of the gastric cancer cells. Although the primary breast cancer was initially misdiagnosed as scirrhous carcinoma because the tumor cells were associated with a dense connective tissue in the stroma, replacement of tumor cells with connective tissue was likely caused by the neoadjuvant chemotherapy.

**Figure 4:**
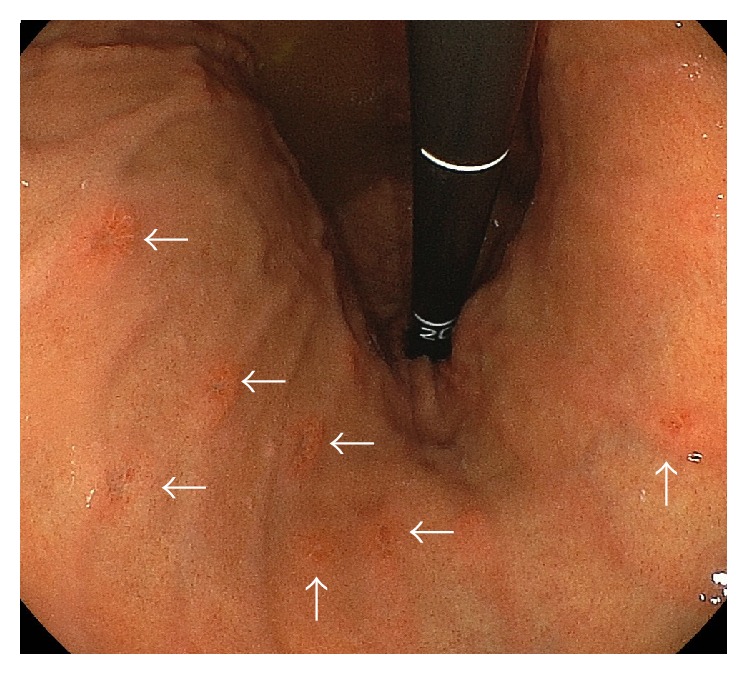
Multiple recurrence was documented at 40 months after the endoscopic treatment.

The endoscopic submucosal dissection appeared to have completed removing the metastatic tumor, and esophagogastroduodenoscopy, computed tomography, and bone scintigraphy were used to confirm that there were no other metastases throughout the patient's body. The patient was subsequently treated with anastrozole, although we detected multiple metastatic tumors at 40 months after the endoscopic treatment. These tumors were located in the gastric body (Figure 4, arrows), and local recurrence was documented in the endoscopically treated area.

## 3. Discussion

It is uncommon for the gastrointestinal tract to be the first site for breast cancer metastasis, as McLemore et al. found that only 41 of 12,001 patients with breast cancer (0.3%) exhibited metastasis in the gastrointestinal tract [1]. Diffuse infiltration into the stomach wall is a representative form of gastric metastasis from breast cancers, which is referred to as the linitis plastica type [4–6]. This type of metastasis is generally visualized via radiography as a rigid and thickened gastric wall, and the macroscopic features can include enlarged mucosal folds, erosions, and/or polypoid lesions [5, 7, 8]. However, metastasis from a breast tumor can also appear as a localized lesion in the stomach, which can mimic early-stage gastric cancers, and present as flat elevated, erosive, ulcerative, or polypoid lesions [3, 7–9].

Invasive ductal carcinoma is the most common type among all patients with breast cancer. However, invasive ductal carcinoma is less frequent among metastatic tumors in the gastrointestinal tract, and invasive lobular carcinoma is the predominant type. Taal et al. have reported 51 patients with gastric metastases from breast carcinoma, which included lobular breast carcinoma (*n* = 36; 70.6%), ductal carcinoma (*n* = 10; 19.6%), and other types (*n* = 5; 9.8%) [10]. McLemore et al. have also reported that 73 patients with 81 breast cancers experienced gastrointestinal metastasis [1] and that 44 of the breast cancers (54.3%) were invasive lobular carcinoma. Interestingly, metastatic tumors from invasive lobular breast cancer occasionally exhibit signet ring cells in the pathological findings, which we also observed in the present case. This issue may mislead clinicians and pathologists and potentially result in a misdiagnosis of primary gastric cancer [11].

To reach an accurate diagnosis, immunostaining is recommended to differentiate between primary gastric cancer and breast cancer metastasis to the stomach [3, 5]. Immunostaining for ER and PgR has been reported to be useful for diagnosing metastatic tumors from breast cancer, although 32% and 12% of primary gastric cancer cases are positive for ER and PgR, respectively. Moreover, metastases from breast carcinoma may exhibit negative ER and PgR staining, even if the primary breast cancer is positive for ER and PgR [5, 12]. Therefore, immunostaining for ER and PgR may not be able to definitively differentiate between breast cancer metastases and primary gastric cancers. Immunostaining for mammaglobin and GCDFP-15 may be more practical for identifying breast cancers, because these markers are sensitive and specific for cells that originate from the mammary glands. Therefore, staining for ER, PgR, mammaglobin, and GCDFP-15 may provide more definitive information that can be used to reach the correct diagnosis.

In the present case, the gastric tumor was initially diagnosed as a primary gastric cancer, based on its macroscopic and histopathological features, which included a signet cell-like appearance. Unfortunately, the final diagnosis of a metastatic tumor from breast cancer was only made after pathological evaluation of an endoscopically resected specimen. Thus, cautious evaluation of the gastric epithelium in the biopsied specimen might have provided a diagnostic clue that could have indicated more exhaustive immunostaining for breast cancer markers. Therefore, the possibility of gastric metastasis from breast cancer should be considered if a patient presents with a gastric lesion and a history of breast cancer. Moreover, it is vital to distinguish between breast cancer metastasis to the stomach and primary gastric cancer, because treatment for the metastatic tumor usually involves systemic therapy, rather than a local treatment (e.g., surgical resection) for gastric lesions.

In summary, we experienced a case of invasive lobular breast carcinoma with a metastatic gastric tumor. However, the endoscopic and histopathological features of the metastatic gastric lesion were very similar to those of primary gastric cancer. Therefore, despite the low prevalence of gastric metastasis, physicians should consider the possibility of gastrointestinal metastasis when gastrointestinal tumors are identified in patients with a history of breast cancer.
